# YTHDF2 destabilizes m^6^A-containing RNA through direct recruitment of the CCR4–NOT deadenylase complex

**DOI:** 10.1038/ncomms12626

**Published:** 2016-08-25

**Authors:** Hao Du, Ya Zhao, Jinqiu He, Yao Zhang, Hairui Xi, Mofang Liu, Jinbiao Ma, Ligang Wu

**Affiliations:** 1 State Key Laboratory of Molecular Biology, National Center for Protein Science Shanghai, Institute of Biochemistry and Cell Biology, Shanghai Institutes for Biological Sciences, University of Chinese Academy of Sciences, Chinese Academy of Sciences, 320 Yue Yang Road, Shanghai 200031, China; 2 CAS-Shanghai Science Research Center, Chinese Academy of Sciences, Shanghai 201204, China; 3 Shanghai Key Laboratory of Molecular Andrology, State Key Laboratory of Molecular Biology, Institute of Biochemistry and Cell Biology, Shanghai Institutes for Biological Sciences, Chinese Academy of Sciences, Shanghai 200031, China; 4 State Key Laboratory of Genetic Engineering, Collaborative Innovation Center of Genetics and Development, Department of Biochemistry, Institute of Plant Biology, School of Life Sciences, Fudan University, Shanghai 200433, China; 5 Shanghai Institute of Planned Parenthood Research, Shanghai 200032, China; 6 School of Life Sciences, Shanghai University, 333 Nanchen Road, Shanghai 200444, China

## Abstract

Methylation at the *N*6 position of adenosine (m^6^A) is the most abundant RNA modification within protein-coding and long noncoding RNAs in eukaryotes and is a reversible process with important biological functions. YT521-B homology domain family (YTHDF) proteins are the readers of m^6^A, the binding of which results in the alteration of the translation efficiency and stability of m^6^A-containing RNAs. However, the mechanism by which YTHDF proteins cause the degradation of m^6^A-containing RNAs is poorly understood. Here we report that m^6^A-containing RNAs exhibit accelerated deadenylation that is mediated by the CCR4–NOT deadenylase complex. We further show that YTHDF2 recruits the CCR4–NOT complex through a direct interaction between the YTHDF2 N-terminal region and the SH domain of the CNOT1 subunit, and that this recruitment is essential for the deadenylation of m^6^A-containing RNAs by CAF1 and CCR4. Therefore, we have uncovered the mechanism of YTHDF2-mediated degradation of m^6^A-containing RNAs in mammalian cells.

## Abstract (web summary)

The YTHDF family of proteins are able to bind and regulate the stability of methylated N6 RNA. Here the authors show that this decreased m^6^A RNA stability is mediated by direct recruitment of the CCR4–NOT deadenylase complex through YTHDF proteins.

## Graphical abstract

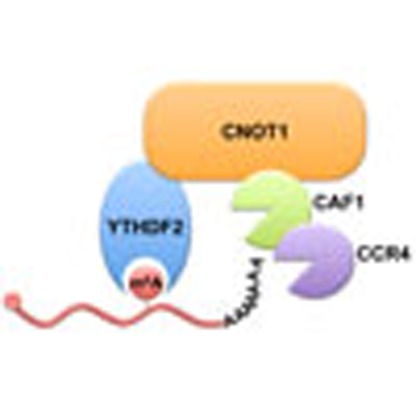

It is well-known that chemical modifications occur on genomic DNA and histone proteins, and that these modifications affect gene expression and have critical biological functions^1^,^2^,^3^,^4^. RNA can also be modified in various ways, although much less is known regarding the mechanisms and functions of RNA modifications^5^. Among them, methylation at the *N*6 position of adenosine (m^6^A) is the most abundant RNA modification within messenger RNAs and long noncoding RNAs (lncRNAs) in many eukaryotic species, including yeast and mammals. Transcriptome analyses of human cells and mouse tissues have revealed that m^6^A modifications are often enriched within an RR(m^6^A)CH sequence motif (where R refers to G or A and H refers to U, A or C) of RNAs^6^,^7^, and that, on average, each RNA contains approximately three to five m^6^A modifications^8^. In mammalian cells, m^6^A is generated in the nucleus by METTL3/METTL14/WTAP, a multicomponent methyltransferase complex^9^,^10^,^11^, and is removed by demethylase FTO and/or ALKBH5 (refs 12, 13). These writer and eraser proteins mainly exist in the nucleus and co-localize with nuclear speckles. The reversible nature of m^6^A modification suggests its importance in biological functions. Indeed, accumulating evidence indicates fundamental regulatory roles of m^6^A modification in a variety of biological processes. In mammals, m^6^A is reported to regulate self-renewal and differentiation of embryonic stem cells^14^,^15^,^16^,^17^, mouse fertility^13^, dopaminergic midbrain circuitry^18^ and the circadian clock^19^. In lower eukaryotic organisms, m^6^A also plays major regulatory roles such as regulating meiosis in yeast^20^, oogenesis in flies^21^ and multiple developmental processes in zebrafish^11^.

Despite the important biological functions of m^6^A in species from yeast to mammals, the molecular mechanism of how m^6^A executes its regulatory roles is still poorly understood. Accumulating evidences suggest that m^6^A regulates the stability, processing or translation of the modified RNA mainly through the recruitment of specific reader proteins or through changing RNA structure. The YT521-B homology domain family (YTHDF) proteins are cytoplasmic readers of m^6^A that selectively recognize and bind to m^6^A within the consensus RR(m^6^A)CH sequence^6^,^22^. Human YTH domain family proteins include three members, YTHDF1–3, each of which comprise a highly conserved single-stranded RNA-binding domain located at the carboxy terminus (the YTH domain)^23^ and a less conserved amino-terminal region. The crystal structure of the YTH domain of human YTHDF2 reveals an aromatic cage that specifically recognizes m^6^A (refs 24, 25). Binding YTHDF2 would thus target m^6^A-containing RNAs to cytoplasmic decay sites for degradation^22^. m^6^A also destabilizes some transcripts indirectly through its effects on HuR binding and microRNA (miRNA) targeting^16^. In the nucleus, binding of the m^6^A reader protein YTHDC1 or HNRNPA2B1 regulates mRNA splicing^26^ or promotes primary miRNA processing, respectively^27^. Moreover, m^6^A regulates mRNA abundance and alternative splicing indirectly by remodeling RNA structure^28^. In addition, recent studies have suggested that the m^6^A reader YTHDF1, the m^6^A writer METTL3 and even m^6^A modification itself can promote the translation of mRNAs^29^,^30^,^31^. These observations suggest that m^6^A regulates many aspects of gene expression via multiple mechanisms.

The regulation of RNA stability is a critical step in the control of RNA metabolism. Normally, eukaryotic mRNAs and lncRNAs are protected by a 5′-cap structure and a 3′-poly-adenine (poly(A)) tail. On receiving a degradation signal, the RNA molecule is subjected to one of the following pathways: the deadenylation-dependent decay pathway that starts with the shortening of the poly(A) tail, the deadenylation-independent decay pathway that starts with the removal of the 5′-cap structure or the endonuclease-mediated decay pathway that is initiated by internal cleavage of the RNA. All of these pathways will target unprotected RNAs for rapid degradation at both ends by the 5′- and 3′-exonucleases^32^. Among these pathways, the deadenylation-dependent decay pathway is used by the bulk of mRNAs in eukaryotes and has been shown to participate in many regulatory pathways. For example, important *trans*-acting regulatory molecules, such as miRNAs^33^,^34^,^35^, and *cis*-acting regulatory sequences, such as AU-rich elements^36^,^37^,^38^,^39^,^40^, both exert their functions by promoting target RNA deadenylation. Deadenylation is triggered by deadenylases, which include the CCR4–NOT complex, the PAN2–PAN3 complex and PARN in mammals. The CCR4–NOT complex is a nine-subunit complex containing two deadenylase subunits, CAF1 (or its paralogue POP2) and CCR4A (or its paralogue CCR4B)^41^. The PAN2–PAN3 complex is a heteromultimer in which PAN2 is the catalytically active deadenylase subunit^42^. PARN is a homodimer^43^. Examples that are subjected to decapping-initiated decay and endonuclease-mediated decay include a subpopulation of mRNAs in yeast^44^,^45^ and nonsense-mutant RNAs in flies^46^,^47^. Previous studies have shown that m^6^A often destabilizes the m^6^A-containing RNAs; however, shortening of the poly(A) tails of RNAs was not observed, posing the possibility that decay of the m^6^A-containing RNA may start by 5′-decapping or endo-cleavage.

In this study, we investigate the degradation mechanism of RNAs bearing m^6^A modifications using a well-established RNA decay monitoring system in mammalian cells. We present evidence that the m^6^A reader protein YTHDF2 recruits the CCR4–NOT deadenylase complex by directly interacting with the superfamily homology (SH) domain of CNOT1, the scaffolding subunit of the complex, to initiate deadenylation and decay of m^6^A-containing mRNAs.

## Results

### m^6^A modification promotes deadenylation of RNAs

**Figure 1:**
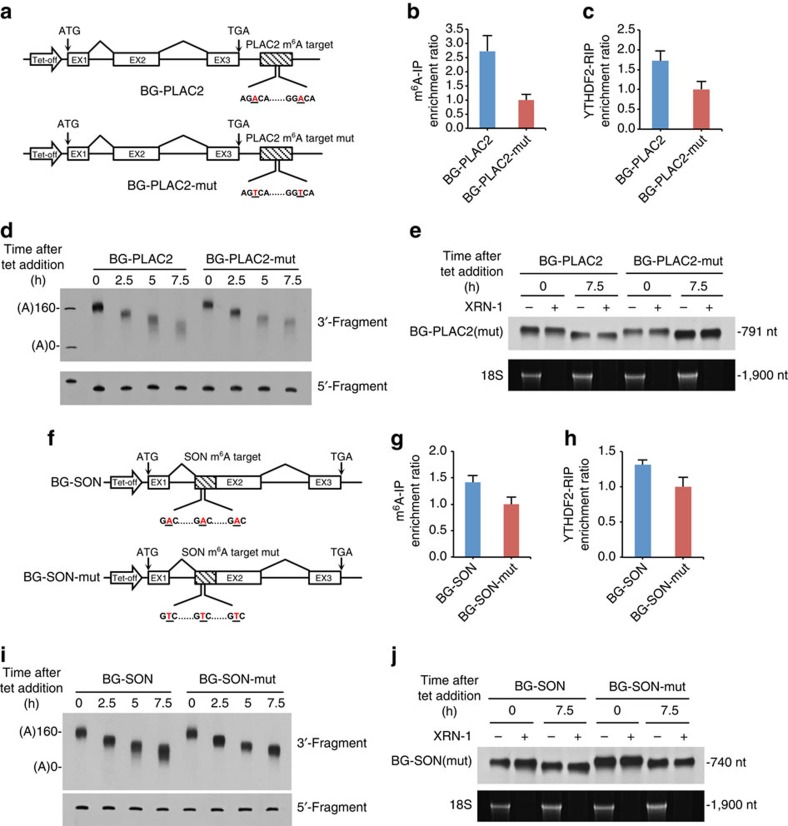
m^6^A modification promotes deadenylation of RNAs. (**a**) The reporter constructs of BG-PLAC2 and BG-PLAC2-mut. A 135-nt fragment from lncRNA *PLAC2* was inserted into the 3′-UTR of the BG reporter (BG-PLAC2). Blank box indicate the open reading frame (ORF). Striped box indicate the inserted DNA fragment. BG-PLAC2-mut is identical to BG-PLAC2, except that the two adenosines (underlined) within the m^6^A motifs were mutated to thymidines. (**b**) The m^6^A-IP enrichment ratio of BG-PLAC2 relative to BG-PLAC2-mut. (**c**) The YTHDF2-RIP enrichment ratio of BG-PLAC2 relative to BG-PLAC2-mut. The RIP assay was performed using anti-FLAG M2 antibody with HeLa-tTA cells that stably express FLAG-tagged YTHDF2. (**d**) Deadenylation assay of BG-PLAC2 and BG-PLAC2-mut. The brief removal of tetracycline (tet) from the culture medium generated a homogenous population of BG mRNAs that underwent synchronous decay. RNA samples were collected at the indicated time intervals and subjected to site-specific cleavage by RNase H to produce 3′- and 5′-BG mRNA fragments. RNA samples were then separated by electrophoresis and detected by northern blotting. (**e**) Retention of the 5′-cap on BG-PLAC2 and BG-PLAC2-mut undergoing deadenylation. The BG-PLAC2 and BG-PLAC2-mut RNA samples collected at 0 and 7.5 h from **d** were treated or not treated with the 5′-phosphate-dependent exonuclease XRN-1. 18S rRNA, which lacks a 5′-cap, served as a positive control for XRN-1 activity. (**f**–**j**) The same experiments as in **a**–**e** for BG-SON and BG-SON-mut. Error bars, mean±s.d., *n*=3, biological replicates.

The m^6^A modification is known to destabilize the RNA carrying it; however, the decay pathway of m^6^A-containing RNAs is not yet known. To address this question, we used a transiently inducible β-globin (BG) reporter system and a transcriptional pulse-chase assay, to investigate the effect of m^6^A modification on RNA degradation^48^. In brief, briefly removing tetracycline (tet) from the culture medium generated a homogenous population of BG mRNA that underwent synchronous decay. RNA samples were then collected at different time points and subjected to site-specific cleavage by RNase H to produce 3′- and 5′-BG mRNA fragments that facilitate a more accurate measurement of the poly(A) tail length via high-resolution gel electrophoresis followed by northern blotting. A fragment containing two m^6^A motifs from *PLAC2*, an m^6^A-containing lncRNA validated by both m^6^A-immunoprecipitation (m^6^A-IP) and YTHDF2 PAR-CLIP assays^22^, was inserted into the 3′-untranslated region (UTR) of the BG reporter (BG-PLAC2). In the control reporter BG-PLAC2-mut, the two adenosines within the two adjacent m^6^A motifs were mutated to thymidine (Fig. 1a). The BG-PLAC2 was significantly enriched compared with the BG-PLAC2-mut according to the immunoprecipitation assays with m^6^A- (Fig. 1b) or YTHDF2-specific (Fig. 1c) antibodies, indicating that these two mutated adenosines were indeed methylated and were recognized by YTHDF2 in the cells. The northern blotting of the 3′-fragments of the RNAs showed that shortening of the poly(A) tail of BG-PLAC2 was accelerated compared with BG-PLAC2-mut (Fig. 1d). In contrast, the length of the 5′-fragments of both the BG reporter RNAs remained intact, indicating that the decay did not start at the 5′-end. We further treated RNA samples with the 5′-exonuclease XRN-1 and found that BG-PLAC2 RNAs undergoing deadenylation still retained their 5′-caps, even at a later time point when the poly(A) tail of the RNA was significantly shortened (Fig. 1e). Moreover, no intermediates of RNA fragments generated by endonuclease cleavage were observed. These results suggest that the decay of m^6^A-containg RNA is initiated by deadenylation.

Previous transcriptome studies have shown that many mRNAs contain multiple m^6^A sites in the open reading frame (ORF)^22^. To investigate whether m^6^A located in the ORF can also mediate RNA deadenylation, we inserted an 84-nt fragment derived from the ORF of *SON* mRNA, which harbours three consensus m^6^A motifs, in-frame into exon 2 of the BG reporter (BG-SON). In the control reporter, the three adenosines within the m^6^A motifs were mutated to thymidines (BG-SON-mut) (Fig. 1f). Both m^6^A-IP and YTHDF2-RIP verified the presence of m^6^A modifications in the wild-type BG-SON reporter mRNA (Fig. 1g,h). Interestingly, BG-SON also exhibited accelerated deadenylation (Fig. 1i) without obvious decapping events (Fig. 1j) compared with BG-SON-mut. These observations demonstrate that m^6^A modification in the ORF or the 3′-UTR of RNAs can promote deadenylation of those RNAs in mammalian cells.

### YTHDF2 destabilizes mRNA by hastening deadenylation

**Figure 2:**
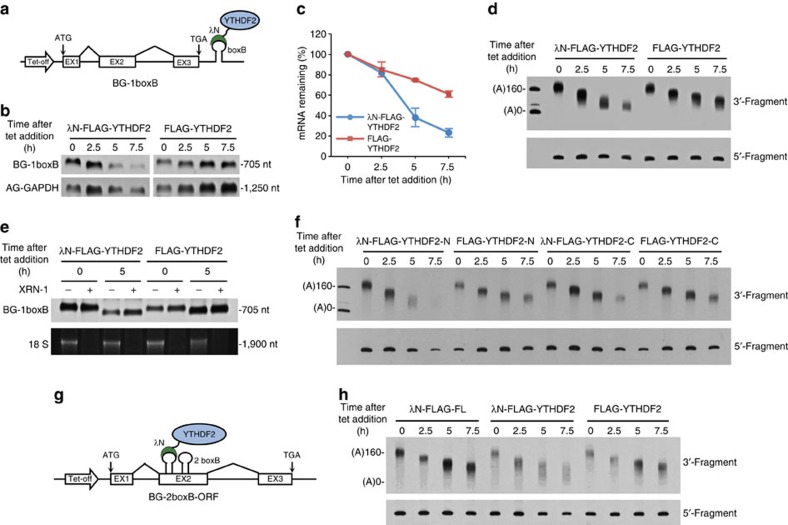
YTHDF2 destabilizes mRNA by hastening deadenylation as an initial step. (**a**) Construct of BG-1boxB, which can bind to proteins fused with a λN peptide through the boxB sequence. (**b**) Decay of BG-1boxB mRNA in the presence of λN-FLAG-YTHDF2 or FLAG-YTHDF2. AG-GAPDH, a constitutively transcribed internal standard was co-transfected. (**c**) Graphs of the concentration of BG mRNA described in **b** as a function of time. The abundance of BG-1boxB mRNA remaining at each time point was normalized to that of AG-GAPDH. Error bars, mean±s.d., *n*=3, biological replicates. (**d**) Deadenylation assay of BG-1boxB mRNA in the presence of λN-FLAG-YTHDF2 or FLAG-YTHDF2. (**e**) Retention of the 5′-cap on BG-1boxB mRNA in the presence of λN-FLAG-YTHDF2 or FLAG-YTHDF2. (**f**) Deadenylation assay of BG-1boxB mRNA tethered with an N-terminal P/Q/N-rich region (YTHDF2-N) or a C-terminal YTH domain (YTHDF2-C). (**g**) Construct of BG-2boxB-ORF, which has two boxB sequences inserted in-frame to the ORF of the BG reporter. (**h**) Deadenylation assay of BG-2boxB-ORF mRNA in the presence of λN-FLAG-FL, λN-FLAG-YTHDF2 or FLAG-YTHDF2.

We next sought to determine whether YTHDF proteins, the cytoplasmic readers of m^6^A, are sufficient to mediate the accelerated deadenylation and decay of the RNA to which they bind. Human YTHDF2 was fused to the λN peptide (λN-FLAG-YTHDF2), which binds to a boxB sequence in the RNA with high affinity^49^ (Fig. 2a). Tethering YTHDF2 to the BG reporter with a single copy of boxB (BG-1boxB) markedly accelerated its deadenylation (Fig. 2d) and decay (Fig. 2b,c) rates compared with the non-tethering control. By measuring the poly(A) tail length and mRNA abundance at each time point, we found that the majority of BG-1boxB mRNA did not decay until the poly(A) tails reached their shortest length. XRN-1 treatment revealed that BG-1boxB mRNA undergoing YTHDF2-mediated deadenylation still retained its 5′-cap (Fig. 2e). The accelerated deadenylation mediated by tethered YTHDF2 (Fig. 2d) appeared to be more dramatic than that of BG-PLAC2 (Fig. 1d) or BG-SON reporters (Fig. 1i) containing natural m^6^A motifs, possibly because the λN peptide has a very high affinity (*K*_d_=5.2 nM)^49^ for the boxB RNA element and because λN-FLAG-YTHDF2 is overproduced. In fact, the boxB-containing reporter RNA deadenylates even faster with further increase in the number of boxB elements (Supplementary Fig. 1a). Moreover, for each specific m^6^A motif within an m^6^A-containing RNA, only a small fraction is actually modified^50^ and the m^6^A site may be bound by other proteins, such as YTHDC1 (ref. 26) and METTL3 (ref. 30), which do not mediate deadenylation or do it less efficiently than YTHDF2. These findings indicate that YTHDF2 destabilizes mRNA by hastening deadenylation as an initial step. YTHDF1 and YTHDF3 also promote deadenylation when tethered to BG-1boxB, although to a lesser extent (Supplementary Fig. 1b). Western blotting ruled out the possibility of a lower expression level of YTHDF1 and YTHDF3 in the cells (Supplementary Fig. 1c). We then focused on YTHDF2 for further characterization. YTHDF2 consists of a C-terminal YTH domain (YTHDF2-C) that selectively binds m^6^A and a P/Q/N-rich N-terminal region (YTHDF2-N). We tethered each domain of YTHDF2 to BG-1boxB mRNA and found that tethered YTHDF2-N significantly promoted poly(A) tail shortening, with an effect comparable to the full-length YTHDF2; by contrast, tethering of YTHDF2-C did not induce deadenylation (Fig. 2f and Supplementary Fig. 1d).

As m^6^A occurs on both the 3′-UTR and the ORF of mRNA, we inserted two boxB sequences in-frame into the ORF of the BG mRNA (BG-2boxB-ORF) (Fig. 2g) and found that accelerated deadenylation was only observed when the reporter was tethered with YTHDF2 but not with a control protein firefly luciferase (FL) (Fig. 2h), consistent with the ORF-m^6^A-mediated decay of BG-SON RNA (Fig. 1i). These observations suggest that YTHDF2 recognizes m^6^A located in both the coding and noncoding regions of RNAs through its C-terminal YTH domain and promotes deadenylation through its N-terminal region.

### CCR4–NOT is responsible for YTHDF2-mediated deadenylation

**Figure 3:**
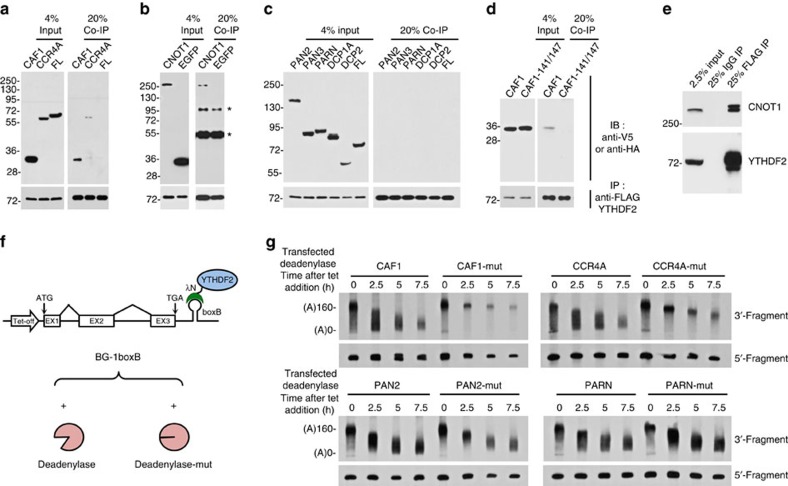
The CCR4–NOT complex is responsible for YTHDF2-mediated RNA deadenylation. (**a**–**c**) Interaction between YTHDF2 and deadenylases or decapping enzymes. HEK 293 cells were co-transfected with a plasmid encoding FLAG-tagged YTHDF2 and either a V5-tagged CAF1, CCR4A, PAN2, PAN3, PARN, DCP1A, DCP2, FL or an HA-tagged CNOT1 or enhanced green fluorescent protein (EGFP). Lysates were subjected to immunoprecipitation by using anti-FLAG affinity gel. Input and co-purified proteins were blotted by probing with corresponding antibodies. Nonspecific bands were indicated by the asterisk. (**d**) Interaction between YTHDF2 and CAF1 or CAF1-141/147, a mutant version of CAF1 with M141K and L147K double substitution. (**e**) Interaction between stably expressed FLAG-tagged YTHDF2 and endogenous CNOT1 in HeLa-tTA cells. (**f**) Strategy of screening of functional deadenylases that are responsible for YTHDF2-mediated deadenylation. BG-1boxB reporter was co-transfected with λN-FLAG-YTHDF2 and either a wild-type or catalytically inactive deadenylase. (**g**) Deadenylation assay of BG-1boxB tethered by YTHDF2 with either a co-transfected wild-type or catalytically inactive deadenylase.

It has been reported that Pho92, a YTHDF2 homologue in *Saccharomyces cerevisiae*, interacts physically with POP2 (a CAF1 homologue) to recruit the POP2–CCR4–NOT deadenylase complex and reduce mRNA stability^51^. To investigate whether accelerated deadenylation of m^6^A-containing RNAs was achieved by the direct recruitment of deadenylase(s) by YTHDF2 in mammals, we screened for interactions between YTHDF2 and components of the potential cellular deadenylation complexes using co-immunoprecipitation (co-IP) assays. Mammalian cells contain three major types of deadenylases: CCR4–NOT, PAN2–PAN3 and PARN. Plasmids encoding the V5-tagged or HA-tagged subunits of these deadenylase complexes and FLAG-tagged YTHDF2 were co-expressed in human HEK293 cells. Cell extracts were treated with RNase A and then subjected to co-IP using anti-FLAG affinity gel. YTHDF2 co-immunoprecipitated with CAF1, CCR4A and CNOT1 (Fig. 3a, b), but not with PAN2, PAN3 and PARN (Fig. 3c). Moreover, no interaction with the decapping complex components DCP1A or DCP2 was detected (Fig. 3c). The human CCR4–NOT complex is a large complex consisting of nine subunits, including a large scaffold subunit, CNOT1, and two catalytically active exoribonuclease subunits, CAF1 (or its paralogue POP2), which directly binds to CNOT1, and CCR4A (or its paralogue CCR4B), which indirectly binds to CNOT1 through CAF1 (refs 41, 52, 53). The co-IP results showed that CAF1 and CCR4A bind with YTHDF2 less efficiently than CNOT1, suggesting that they may interact with YTHDF2 indirectly. To test this hypothesis, we generated CAF1-141/147 (M141K and L147K double mutant), a mutant form of CAF1 that disrupts the interaction with CNOT1 but retains the ability to interact with CCR4A (Supplementary Fig. 2a,b)^52^,^53^. As shown by co-IP experiments, the CAF1-141/147 mutant lost its ability to interact with YTHDF2 (Fig. 3d), supporting the idea that CAF1 binds to YTHDF2 through CNOT1. We next sought to determine whether endogenous YTHDF2 interacts with CNOT1. Previous studies have identified YTHDF2 targets via FLAG-tagged YTHDF2 due to the lack of a highly specific antibody^22^. We therefore established a HeLa-tTA cell line that stably expresses FLAG-tagged YTHDF2 at near-endogenous levels (Supplementary Fig. 2c) and performed a co-IP with an antibody against the FLAG tag. Consistent with co-IP results obtained for overexpressed proteins, we found that endogenous CNOT1 is efficiently co-immunoprecipitated with FLAG-tagged YTHDF2 (Fig. 3e). To further confirm the hypothesis that CAF1 and CCR4 are the ribonucleases responsible for the YTHDF2-mediated deadenylation, we analysed the dominant-negative effect of each inactive deadenylase on the deadenylation of BG-1boxB mRNA tethered with λN-FLAG-YTHDF2 (Fig. 3f). Overproducing catalytically inactive CAF1 or CCR4A significantly impaired the poly(A) shortening of BG-1boxB tethered with YTHDF2, whereas PAN2-mut and PARN-mut exhibited no obvious effects (Fig. 3g and Supplementary Fig. 2d), suggesting that CAF1 and CCR4A are the ribonucleases responsible for YTHDF2-mediated deadenylation through the recruitment of CNOT1 by YTHDF2.

### The SH domain of CNOT1 directly interacts with YTHDF2

**Figure 4:**
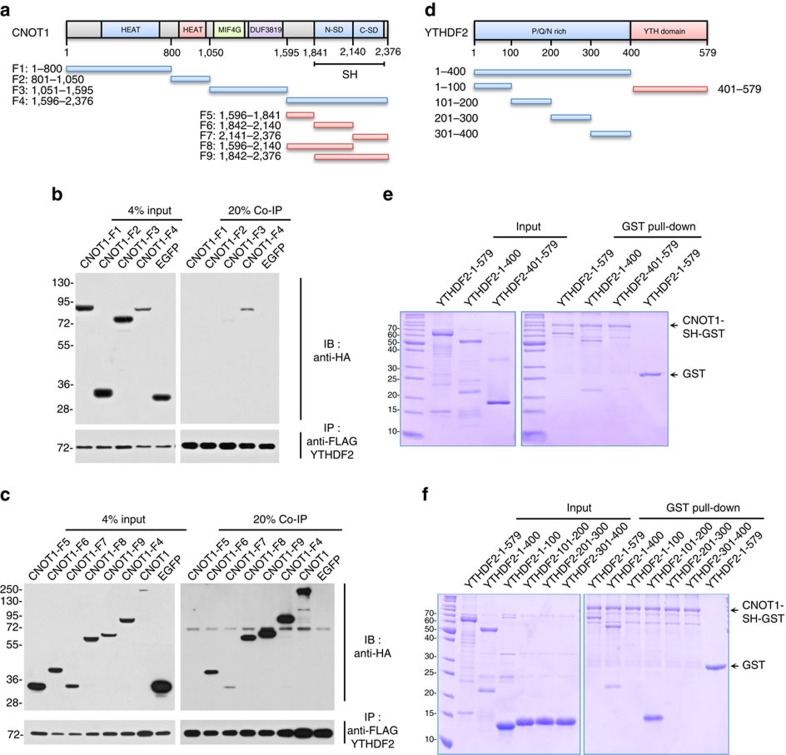
The SH domain of CNOT1 directly interacts with YTHDF2. (**a**) Schematic diagram of human CNOT1 and the fragments used in **b** and **c**. (**b**,**c**) Interaction between YTHDF2 and CNOT1 fragments. HEK 293 cells were co-transfected with a plasmid encoding FLAG-tagged YTHDF2 and an HA-tagged CNOT1 fragment or a negative control (EGFP). Immunoprecipitation and immunoblotting were performed as in Fig. 3a–c. (**d**) Schematic diagram of human YTHDF2 and the fragments used in **e** and **f**. (**e**,**f**) Determination of a direct interaction between CNOT1-SH and YTHDF2 fragments by GST pull-down assays. Recombinant His-tagged YTHDF2 or its fragments and GST-tagged CNOT1-SH were purified, and equal amounts of both proteins were subjected to GST pull-down assay followed by SDS–PAGE separation and Coomassie Blue staining.

To identify the domain of CNOT1 interacting with YTHDF2, we divided CNOT1 into four fragments (Fig. 4a) and analysed the interactions of each fragment with YTHDF2 using co-IP assays. Among fragments F1–F4, only CNOT1-F4 was co-purified with YTHDF2 (Fig. 4b). CNOT1-F4 contains an undefined region followed by the NOT1 SH domain, which consists of N- and C-terminal subdomains^54^. We made a further series of truncated fragments of CNOT1-F4 (F5-F9, Fig. 4a) and tested their interactions with YTHDF2. All of the fragments that contain either or both of the N- and C-terminal subdomains were co-immunoprecipitated with YTHDF2 (Fig. 4c). Importantly, CNOT1-F9, which contains the full-length SH domain, exhibited the strongest interaction, suggesting that the SH domain mediates the interaction of CNOT1 with YTHDF2.

To investigate whether the interaction between the CNOT1 SH domain and YTHDF2 is direct, we performed glutathione *S*-transferase (GST) pull-down assays with purified recombinant proteins from *Escherichia coli* (Fig. 4d). The CNOT1 SH domain–GST fusion protein (CNOT1-SH-GST) efficiently pulled down full-length YTHDF2 at molecular ratio near 1:1 (Fig. 4e), confirming a direct and stable interaction. CNOT1-SH-GST also efficiently pulled down YTHDF2-N (YTHDF2-1-400) but not YTHDF2-C (YTHDF2-401-579) (Fig. 4e), which is consistent with previous deadenylation results using the tethering system (Fig. 2f). We further divided YTHDF2-N into four regions (aa 1–100, aa 101–200, aa 201–300 and aa 301–400) (Fig. 4d) and found that only YTHDF2-101-200 was pulled down by CNOT1-SH-GST (Fig. 4f). These observations indicate that YTHDF2 recruits the CCR4–NOT complex through a direct interaction between its N terminus and the SH domain of CNOT1.

### CNOT1 recruitment is essential for m^6^A RNA degradation

**Figure 5:**
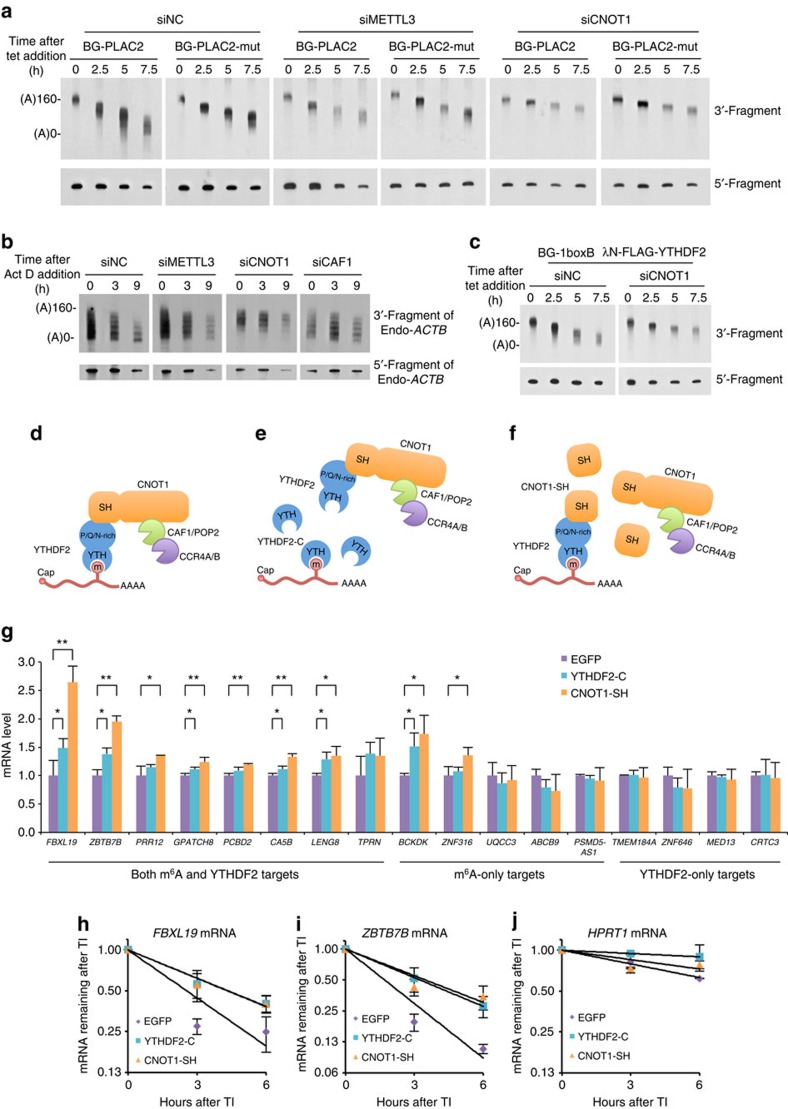
Recruitment of CCR4–NOT through CNOT1 is essential for the degradation of endogenous m^6^A-containing RNAs. (**a**) Deadenylation assay of BG-PLAC2 and BG-PLAC2-mut on knocking down of endogenous METTL3 or CNOT1 by siRNA. siNC served as a negative control. (**b**) Deadenylation assay of endogenous *ACTB* mRNA on knocking down of endogenous METTL3, CNOT1 or CAF1 by siRNA. (**c**) Deadenylation assay of BG-1boxB mRNA in the presence of λN-FLAG-YTHDF2 on knocking down of endogenous CNOT1 by siRNA. (**d**–**f**) Schematic diagrams of YTHDF2-mediated deadenylation of m^6^A-containing RNAs and the dominant-negative effect of YTHDF2-C or CNOT1-SH. m^6^A is recognized by YTHDF2, which further recruits the CCR4–NOT complex by interacting with CNOT1 (**d**). Overexpressed YTHDF2-C occupies m^6^A sites but is incapable of recruiting the CNOT1 subunit of the CCR4–NOT complex; thus, it impairs the deadenylation and decay of m^6^A-containing RNAs (**e**). Overexpressed CNOT1-SH binds to endogenous YTHDF2 but is incapable of recruiting the catalytic subunits CAF1 and CCR4A/B; thus, it impairs the deadenylation and decay of m^6^A-containing RNAs (**f**). (**g**) Expression level of endogenous target mRNAs measured by quantitative reverse transcriptase–PCR on overexpression of YTHDF2-C, CNOT1-SH or the negative control green fluorescent protein (EGFP). All values were normalized to *HPRT1* mRNA, a housekeeping gene previously reported to contain no m^6^A modifications and is not bound by YTHDF2. Error bars, mean±s.d., *n*=3, biological replicates. **P<0.01, *P<0.05, *t*-test. (**h**–**j**) Half-life of *FBXL19*, *ZBTB7B* and *HPRT1* mRNA on overexpression of YTHDF2-C, CNOT1-SH or the negative control EGFP after transcription inhibition (TI). All values were normalized to 18S rRNA. Error bars, mean±s.d., *n*=3, biological replicates.

To confirm that the deadenylation events that we observed are caused by m^6^A and mediated by the CCR4–NOT complex, we knocked down METTL3, the writer of m^6^A, and CNOT1, the bridge between the m^6^A reader and the deadenylase, and performed deadenylation assays. Compared with siNC, knocking down METTL3 reduced the deadenylation rate of BG-PLAC2, a reporter RNA that contains wild-type m^6^A motifs, but did not affect the deadenylation rate of BG-PLAC2-mut, a reporter RNA that contains mutant m^6^A motifs (Fig. 5a). This suggests that the accelerated deadenylation of BG-PLAC2 is indeed caused by m^6^A modification. In addition, knocking down CNOT1 significantly reduced the deadenylation rate of both BG-PLAC2 and BG-PLAC2-mut (Fig. 5a), possibly because the CCR4–NOT complex is a common effector of multiple RNA degradation pathways. Similar results were obtained using the BG-1boxB and λN-FLAG-YTHDF2 tethering system (Fig. 5c). Next, we wanted to determine whether the deadenylation of an endogenous m^6^A-containing RNA is regulated by the CCR4–NOT complex. *ACTB* mRNA, which is a YTHDF2-PAR-CLIP target^22^, has a relatively high m^6^A modification rate (>20%) at one motif^50^ and is readily detected by northern blotting, making it an ideal candidate for further characterization. Actinomycin D was added to the cell culture medium to terminate transcription and RNAs were collected at different time points to monitor the deadenylation rate of endogenous *ACTB* mRNA. A slower rate of *ACTB* mRNA poly(A) shortening was observed on knocking down METTL3, CNOT1 or CAF1, a catalytic component of the CCR4–NOT complex (Fig. 5b). These results support the hypothesis that CNOT1 is essential for m^6^A-mediated deadenylation.

To further validate that the interaction between YTHDF2 N terminus and the SH domain of CNOT1 is crucial to the degradation of endogenous m^6^A-containing RNAs (Fig. 5d), we expressed the truncated form of YTHDF2 or CNOT1 in the cells to disrupt the recruitment of the CCR4–NOT complex to m^6^A-containing RNAs by YTHDF2. Overexpressed YTHDF2-C is able to occupy most of the m^6^A sites, but is incapable of interacting with CNOT1; thus, it will not recruit the CCR4–NOT complex (Fig. 5e). Overexpressed CNOT1-SH will compete with endogenous CNOT1 for binding to YTHDF2, but is incapable of recruiting the catalytically active subunits CAF1 and CCR4 (Fig. 5f and Supplementary Fig. 3a). Therefore, both of the truncated proteins should exert dominant-negative effects on the degradation of endogenous m^6^A-containing RNAs. We measured the changes in abundance of RNAs bearing m^6^A modifications^55^ as well as those bound with YTHDF2 (ref. 22) on the overexpression of truncation mutants in HeLa-tTA cells. The expression levels of five out of the eight transcripts bearing m^6^A modification and bound with YTHDF2 were significantly increased (Fig. 5g and Supplementary Fig. 3b). CNOT1-SH exhibited an overall stronger dominant-negative effect than YTHDF2-C, possibly because the degradation of these transcripts also relies on additional CCR4–NOT-dependent deadenylation pathways, such as miRNA-mediated mRNA deadenylation by AGOs and GW182. We also measured the half-lives of the two most upregulated RNAs among the five targets and confirmed that these RNAs became more stable (Fig. 5h,i) compared with the non-m^6^A target *HPRT1* (Fig. 5j). Moreover, overexpression of either YTHDF2-C or CNOT1-SH reduced the deadenylation rate of *PLAC2* RNA expressed from a minigene reporter (Supplementary Fig. 4). In contrast, only two out of the five m^6^A-only targets exhibited significantly elevated RNA levels (Fig. 5g). Notably, the m^6^A-only targets also showed an overall weaker m^6^A signal than targets with both m^6^A modification and YTHDF2 binding^22^,^55^. It therefore seems that most m^6^A-containing RNAs are bound by YTHDF2 and subjected to accelerated deadenylation mediated by CCR4–NOT. Interestingly, none of the four YTHDF2-only targets increased their expression levels on the overexpression of YTHDF2-C or CNOT1-SH (Fig. 5g), in seeming contradiction with the result of the YTHDF2 tethering assay. Previous study has found that YTHDF2 can bind to the RRACH motif without m^6^A modification, but with a much weaker binding strength compared with the RR(m^6^A)CH motif^22^. Thus, the transient interactions of some RNAs with YTHDF2 that are captured by the PAR-CLIP assay might not be sufficient to stably recruit the CCR4–NOT complex and to trigger the deadenylation and decay of these RNAs. In contrast, in the tethering assay, the λN-fused YTHDF2 protein strongly associated with the boxB-containing reporter RNA and induced efficient deadenylation and decay. The other possible explanation for this discrepancy is that the recognition of m^6^A modification by the RRACH motif may induce a conformational change of YTHDF2, thereby enhancing its ability to mediate RNA degradation. Taken together, these results provide evidence that the recruitment of the CCR4–NOT complex through the interaction of the N-terminal region of YTHDF2 and the SH domain of CNOT1 is essential for the YTHDF2-mediated degradation of m^6^A-containing RNAs.

## Discussion

In this study, we have demonstrated that expedited poly(A) shortening is the initiation step of the decay of m^6^A-containing RNAs, and that CAF1 and CCR4A/B of the CCR4–NOT complex are the key deadenylases responsible for the accelerated deadenylation. Moreover, we have shown that the CCR4–NOT complex is recruited to m^6^A-containing RNAs through a direct interaction between the N-terminal region of YTHDF2, the reader of m^6^A and the SH domain of CNOT1, the scaffolding subunit of the CCR4–NOT complex. In a previous study^22^, although tethering the N-terminal region of YTHDF2 to the 3′-UTR of a luciferase reporter led to reduced mRNA levels, no significant change in the deadenylation rate was observed. This discrepancy was most likely observed, because an indirect PCR-based assay was used to measure the poly(A) tail length and the RNA fragments with shorter poly(A) tails tend to be amplified more efficiently, which may mask the differences. Here we employed a transcriptional pulse-chase RNA decay assay and a high-resolution northern blotting assay to monitor the deadenylation rate. We found that naturally occurring m^6^A sequences located in both the ORF and the 3′-UTR could mediate accelerated poly(A) removal in the cells. Tethering m^6^A reader proteins to the reporter RNA recapitulates the phenomenon, suggesting a direct role of YTHDF family proteins in promoting deadenylation of m^6^A-containg RNAs. In contrast to the previous view that binding of YTHDF2 to m^6^A-containing mRNA occurs in parallel or at a later stage of deadenylation^22^, our results indicate that initiation of deadenylation and decay relies on the binding of YTHDF2 to the RNA. Moreover, although multiple nucleases, including the decapping enzyme DCP1A and the deadenylase CAF1, co-localized with YTHDF2 in the P-body of the cells^22^, we found that deadenylation occurred before the removal of the 5′-cap from the m^6^A-containing mRNAs. Intriguingly, GW182, the core component of the P-body, does not exhibit a detectable interaction with YTHDF2 (Supplementary Fig. 5), indicating an indirect role of the P-body in the degradation of m^6^A RNAs. All of these observations support the model that binding of YTHDF2 causes deadenylation and decay of m^6^A-containing RNAs through direct recruitment of the CCR4–NOT complex, although multiple mechanisms, which warrant further investigation, may regulate the stability, export or translation of m^6^A-containing RNAs in the cells.

We discovered two major bands of endogenous CNOT1 that co-immunoprecipitated with YTHDF2, with the larger one being enriched more efficiently. CNOT1 protein is known to have multiple isoforms and posttranslational modifications. We speculated that YTHDF2 may have stronger interaction with the less abundant isoform of CNOT1 that migrated slightly slowly, an interesting observation worthy of future investigation. Our *in vitro* GST pull-down assay has mapped the CNOT1 interaction region in YTHDF2 to its N-terminal aa 101–200; however, tethering a YTHDF2 mutant lacking aa 101–200 to the reporter mRNA still caused some degree of accelerated deadenylation (Supplementary Fig. 5). Given that this region appears to be flexible and lacks a clear domain structure, it is possible that multiple sites across the N-terminal region of YTHDF2 may be cooperatively involved in the interaction with CNOT1 *in vivo*. Further structural studies are needed to reveal the details of such interactions between YTHDF2-N and the SH domain of CNOT1.

## Methods

### Plasmid constructions

The rabbit BG coding region was placed downstream of a tet-off promoter to generate the plasmid pTBG, on which all BG reporters are designed. pBG-PLAC2 was constructed by inserting the *PLAC2* fragment containing two m^6^A motifs (5′-CATCGCAAGAAGAGAAGCACAGAAGGGGCA GGAGAGACACTCAGAGGCACTTCCGCTCTTGCCCAGGACATTTTCCCAGCCACACCTTTGCCCAAGCCGTGCCCCCTGCCTGGAGCACTTTTCAACCTCTTCTCT-3′) into the 3′-UTR of pTBG between the NheI and XbaI sites. pBG-SON was constructed by inserting the *SON* fragment containing three m^6^A motifs (5′-AACACCATGGACTCCCAGATGTTAGCGTCTAGCACCATGGACTCCCAGATGTTAG CAACTAGCTCCATGGACTCCCAGATGTTA-3′) in-frame into exon 2 of pTBG using the NcoI site. pBG-PLAC2-mut and pBG-SON-mut were constructed by mutating the adenosines in the GAC motifs to thymidines. pBG-1boxB and pBG-4boxB were generated by inserting one boxB sequence (5′-GCCCTGAAGAAGGGC-3′) or four boxB sequences (5′-GCCCTGAAAAAGGGCGATCTAGCCCTG AAAAAGGGCTCTAGCGCCCTGAAAAAGGGCGATCTAGCCCTGAAAAAGGGC-3′) into the 3′-UTR of pTBG. pBG-2boxB-ORF was constructed by inserting two boxB sequences (5′-GCCCTGAAAAAGGGCGATCTAGCCCTGAAAAAGGGC-3′) in-frame into exon 2 of the pTBG reporter using the NcoI site. *PLAC2* minigene reporter was constructed by replacing BG coding region in pTBG with the genomic fragment from *PLAC2* gene containing the region from exon 2 to the first 405 bp of exon 3. Plasmids encoding λN-FLAG-YTHDF2/YTHDF1/YTHDF3/YTHDF2-N/YTHDF2-C were constructed by inserting tandem DNA fragments encoding the λN peptide (MNARTRRRERRAEKQAQWKAAN), a FLAG epitope tag (DYKDDDDK), a hexaglycine linker and the corresponding human protein coding region into a pCI vector (Promega). YTHDF2-N (aa 1–389) and YTHDF2-C (aa 390–579) were constructed according to a previous study^22^. Deleting the sequence encoding the λN peptide from the corresponding λN-FLAG plasmids described above generated plasmids encoding FLAG-YTHDF2/YTHDF1/YTHDF3/YTHDF2-N/YTHDF2-C. Plasmids pV5-CAF1, pV5-CCR4A, pV5-PAN2, pV5-PARN, pV5-CAF1-mut, pV5-CCR4A-mut, pV5-PAN2-mut and pV5-PARN-mut have been reported previously^56^. pV5-CNOT1, pV5-PAN3, pV5-DCP1A and pV5-DCP2 were constructed by inserting the corresponding complementary DNA fragments into a pV5 vector. Plasmids expressing all the CNOT1 fragments were constructed by inserting tandem DNA fragments encoding the λN peptide, a haemagglutinin (HA) epitope tag (YPYDVPDYA), a hexaglycine linker and the corresponding CNOT1 fragment into a pCI vector (Promega). For GST pull-down assays, the YTHDF2 fragment was inserted into pET-28a (Novagen) between the BamHI and SalI sites. CNOT1 (aa 1842–2376) was inserted into pGEX-6P-1 (GE Healthcare) between the BamHI and SalI sites. To generate a stable cell line expressing FLAG-tagged YTHDF2, the coding region of YTHDF2 was fused with a C-terminal FLAG-tag and inserted into a modified pGIPZ (Open Biosystems) lentiviral vector.

### Cell culture and stable cell lines

HEK293 and 293T cells were purchased from ATCC. HeLa-tTA cells were purchased from Clontech. All cells were maintained in DMEM medium supplemented with 10% fetal bovine serum (Gibco) and were inspected regularly for mycoplasma contamination. FLAG-tagged YTHDF2-overexpressing HeLa-tTA cells were generated by transduction with lentiviruses in the presence of 8 μg ml^−1^ of Polybrene overnight, followed by a 1-week puromycin selection. To produce the lentiviruses, 293T cells were transfected with a virus vector encoding FLAG-tagged YTHDF2, as well as the VSVG and ΔR8.91 plasmids. Viruses were harvested at 48 and 72 h post transfection.

### m^6^A-IP and YTHDF2-RIP assays

HeLa-tTA cells grown in tetracycline (tet)-free culture medium in 10-cm dish were transfected with 1 μg plasmids of either pBG-PLAC2 or pBG-PLAC2-mut for 24 h. Cytoplasmic RNA was extracted and subjected to m^6^A-IP^57^. Thirty micrograms of RNA was used for each IP reaction in 500 μl IP buffer (10 mM Tris-HCl pH 7.4, 150 mM NaCl, 0.1% NP-40, 0.4 U μl^−1^ RiboLock RNase Inhibitor (Thermo Fisher Scientific) and 2 mM Ribonucleoside vanadyl complexes (New England Biolabs)) with 5 μg of either m^6^A-specific antibody (Synaptic Systems, 202 003) or nonspecific rabbit IgG. Fifty microlitres of pre-washed Protein A argrose (Invitrogen, 15918-014) was added to the IP sample and rotated gently for 2 h at 4 °C. The beads were washed with 1 ml IP buffer for three times and the bound RNA was purified from the beads using Trizol. For YTHDF2-RIP, HeLa-tTA cells that stably expressed FLAG-tagged YTHDF2 in 10-cm dish were transfected with 1 μg plasmids of either pBG-PLAC2 or pBG-PLAC2-mut for 24 h and then harvested in 1 ml lysis buffer (50 mM Tris-HCl pH 7.4, 150 mM NaCl, 1 mM EDTA, 1 mM dithiothreitol, 0.5% NP-40, 0.1 U μl^−1^ RiboLock RNase Inhibitor (Thermo Fisher Scientific) and EDTA-free protease inhibitor cocktail (Roche)). Lysate was placed on ice for 10 min and then centrifuged at 14,000 *g* for 15 min at 4 °C. Five hundred microlitres of supernatant was subjected to 2.5 mg Dynabeads Protein G magnetic beads (Thermo Fisher Scientific, 10004D) coupled with either 5 μg FLAG-M2 antibody (Sigma-Aldrich, F1804) or mouse IgG (Shanghai Immune Biotech, IL005) and rotated for 3 h at 4 °C. Next, the beads were washed with 1 ml lysis buffer three times and RNA was isolated from the beads using Trizol. Reverse transcription and real-time quantitative PCR were used to measure the RNA abundance of BG reporters and *ZBTB7B*, an endogenous m^6^A target, from the input RNA samples and from the m^6^A-IP or YTHDF2-RIP RNA samples. Enrichment ratio of BG-PLAC2 or BG-PLAC2-mut were calculated by normalizing to *ZBTB7B*. PCR primers for BG-PLAC2 and BG-PLAC2-mut are as follows: 5′-TGAGGAGAAGTCTGCGGTCAC-3′ and 5′-GGACTCGAAGAACCTCTGGGT-3′. All the same experiments above were performed for BG-SON and BG-SON-mut, and the PCR primers for BG-SON reporter were as follows: 5′-GCACCTTTGCTAAGCTGAGTG-3′ and 5′-GTATTTGTGAGCCAGGGCATT-3′.

### Deadenylation and decay assays

HeLa-tTA cells were plated on 35-mm plates 1 day before transfection in DMEM containing 20 ng ml^−1^ tet. For deadenylation assays of BG-PLAC2/SON and the corresponding mutants, 500 ng of the reporter plasmid was used for transfection. For YTHDF1-3 and BG-1boxB or BG-2boxB-ORF tethering assays, DNA mixtures containing the BG reporter plasmids (500 ng) and the plasmids encoding the YTHDF proteins (λN-FLAG or FLAG; 200–800 ng) were used for transfection. For testing deadenylase mutants for a dominant-negative phenotype, plasmids encoding a wild-type deadenylase or its catalytically inactive mutant counterpart (pV5-CAF1, pV5-CAF1-mut, pV5-CCR4A, pV5-CCR4A-mut, pV5-PAN2, pV5-PAN2-mut, pV5-PARN and pV5-PARN-mut; 300 ng) were transfected into HeLa-tTA cells together with the BG-1boxB reporter and λN-FLAG- or FLAG-YTHDF2 plasmids. For deadenylation assays on small interfering RNA (siRNA) knockdown, HeLa-tTA cells growing in 60-mm plates were first transfected with 150 pmol siRNA (sequences listed below) using Lipofectamine 2000 transfection reagent (Invitrogen) for 24 h and were then split into 35-mm plates for downstream experiments. The transcription of BG mRNA was induced by removing tet 12 h after transfection. After 3 h of induction, tet was added to a final concentration of 1 μg ml^−1^ to block the transcription of BG. Cytoplasmic RNA was then isolated at various time intervals. Equal amounts of RNA (6 μg) were treated with RNase H in the presence of a DNA oligo (5′-CCAGCCACCACCTTCTGATAGGC-3′ for BG-PLAC2 and BG-PLAC2-mut; 5′-GTCCAGGTGACTCAGACCCTC-3′ for BG-SON, BG-SON-mut, BG-1boxB and BG-2boxB-ORF) complementary to the BG coding region. The digested RNA samples were then analysed by electrophoresis (5.5% PAGE with 8 M urea) and northern blotting using the DIG Northern Starter Kit (Roche). Uncropped scans of the most important northern blottings are shown in Supplementary Fig. 6. For measuring the half-life of BG mRNA, a constitutively transcribed α-globin-GAPDH (AG-GAPDH) mRNA chimera (pSVα1-GAPDH, 100 ng), which served as an internal standard, was co-transfected with each BG reporter. To test for the presence of the 5′-cap, cytoplasmic RNA samples (6 μg) were treated with three units of 5′-phosphate-dependent exonuclease XRN-1 (New England Biolabs) for 2 h at 37 °C, according to the manufacturer's protocol. To detect the deadenylation of endogenous *ACTB* mRNA, actinomycin D was added to the culture medium at a final concentration of 5 mg ml^–1^, to terminate transcription. RNA were collected at 0, 3 and 9 h post transcription termination. RNA samples were treated with RNase H in the presence of a DNA oligo (5′-CTCGCTCCAACCGACTGCTGT-3′) complementary to *ACTB* mRNA. siRNA sequences are as follows:

siMETTL3 S: 5′-GCAAGUAUGUUCACUAUGAAA-3′, AS: 5′-UCAUAGUGAACAUACUUGCAG-3′

S: 5′-GAGCCAGCCAAGAAAUCAAGG-3′, AS: 5′-UUGAUUUCUUGGCUGGCUCCU-3′

siCNOT1 S: 5′-GCUAAAGGAAACGGUGAAAUU-3′, AS: 5′-UUUCACCGUUUCCUUUAGCUU-3′

S: 5′-GUGGACAAUUUAACCAAGAUU-3′, AS: 5′-UCUUGGUUAAAUUGUCCACUU-3′

siCAF1 S: 5′-AACAAGUCUACAUUACACCGC-3′, AS: 5′-GGUGUAAUGUAGACUUGUUAA-3′

S: 5′-ACUCUAACUUGCCUGAAGAUU-3′, AS: 5′-UCUUCAGGCAAGUUAGAGUUU-3′

siNC S: 5′-CGGCAAGCTGACCCTGAAGTT-3′, AS: 5′-AACTTCAGGGTCAGCTTGCCG-3′

### Co-IP assays

HEK 293 cells were transiently co-transfected with plasmids that encode λN-FLAG-YTHDF2 and either V5-tagged deadenylases/DCPs or HA-tagged CNOT1 fragments. At 36 h after transfection, the cells were washed with PBS and harvested in 200 μl lysis buffer (50 mM Tris-HCl pH 7.4, 150 mM NaCl, 1 mM EDTA, 1 mM dithiothreitol, 0.5% NP-40, 0.2 μg ml^−1^ of RNase A (Roche) and EDTA-free protease inhibitor cocktail (Roche)). Lysate was placed on ice for 10 min and clarified by centrifugation at 14,000 *g* for 15 min at 4 °C. The supernatant was then mixed with anti-Flag Affinity gel (Shanghai Genomics, SG4510-10) and incubated for 3 h with gentle rotation at 4 °C. The resin was washed with 1 ml Tris-buffered saline three times and the immunocomplex was eluted with 40 μl Laemmli sample buffer (62.5 mM Tris-HCl (pH 6.8), 2% SDS, 10% (vol vol^−1^) glycerol and 0.002% bromophenol blue) and analysed by electrophoresis and immunoblotting. To co-IP endogenous CNOT1, eight 10 cm dishes of HeLa-tTA cells that stably express FLAG-tagged YTHDF2 reaching 80% confluence were pooled and lysed with 1 ml lysis buffer. Lysate was placed on ice for 10 min and then centrifuged at 14,000 *g* for 15 min at 4 °C. Four hundred microlitres of supernatant was subjected to 2.5 mg Dynabeads Protein G magnetic beads (Thermo Fisher Scientific, 10004D) coupled with either 10 μg FLAG-M2 antibody (Sigma-Aldrich, F1804) or mouse IgG (Shanghai Immune Biotech, IL005) and rotated for 3 h at 4 °C. Beads were washed with 1 ml lysis buffer five times before the immunocomplex was eluted with 40 μl Laemmli sample buffer.

### Immunoblotting assays

Cells lysates or co-IP inputs and elution products were separated on 6–8% polyacrylamide–SDS gels and transferred to a nitrocellulose membrane using transfer buffer (25 mM Tris, 192 mM glycine and 10% methanol). To co-IP endogenous CNOT1, the NuPAGE Novex 4–12% Bis-Tris Gel was used, and the electrophoresis and membrane transfer steps were performed according to the manufacturer's protocol (Thermo Fisher Scientific). The blots were blocked with 5% non-fat milk in PBS containing 0.05% Tween-20 for 30 min and then probed for 1 h at room temperature in PBST with mouse monoclonal anti-FLAG-HRP antibody (1:5,000, Sigma-Aldrich, A8592), mouse monoclonal anti-V5-HRP antibody (1:2,000, Shanghai Genomics, SG4130-40), mouse monoclonal anti-HA-HRP antibody (1:2,000, Shanghai Genomics, SG4130-30), rabbit polyclonal anti-HA antibody (1:2,000, Shanghai Immune Biotech, PA010), rabbit polyclonal anti-YTHDF2 antibody (1:1,000, Abcam, ab170118) or rabbit polyclonal anti-CNOT1 antibody (1:1,000, Proteintech, 14276-1-AP). Next, the blots were incubated with a secondary antibody conjugated to horseradish peroxidase (HRP) (1:5,000, Jackson Immuno Research Laboratories) and detected with an Immun-Star HRP chemiluminescence kit (Bio-Rad). For blots that were probed with anti-FLAG-HRP, anti-V5-HRP or anti-HA-HRP antibodies, no secondary antibody was used. Uncropped scans of the most important western blottings are shown in Supplementary Fig. 6.

### Recombinant protein expression and purification

Full-length and C-terminal fragments of His-tagged recombinant YTHDF2 proteins were expressed in BL21 (DE3) *E. coli* cells and were purified using Ni-NTA resin (GE Healthcare) followed by gel filtration. GST-tagged CNOT1 (aa 1842–2376) was expressed in BL21 (DE3) *E. coli* cells and was purified by glutathione Sepharose-4B. Next, it was further purified over a superdex-G200 size-exclusion column (GE Healthcare) in 20 mM HEPES (pH 7.4) and 200 mM NaCl.

### GST pull-down assays

Each YTHDF2 fragment (40 pmol) was incubated with 30 μl of glutathione Sepharose-4B and 40 pmol of GST or recombinant GST-CNOT1 (aa 1842–2376) in binding buffer (20 mM HEPES (pH 7.4), 200 mM NaCl and 0.1% Tween-20) for 2 h at 4 °C with gentle rocking. Beads were then washed five times with 1 ml binding buffer and proteins were eluted by boiling the beads with 40 μl Laemmli sample buffer at 95 °C for 10 min. Proteins were analysed by SDS–PAGE and visualized by Coomassie Blue staining.

### Real-time quantitative PCR

HeLa-tTA cells were transfected with plasmids encoding either YTHDF2-C, CNOT1-SH or enhanced green fluorescent protein using Lipofectamine 2000 (Invitrogen) for 36 h. Cytoplasmic RNA was extracted and treated with DNase I (Fermentas) and was then reverse transcribed using M-MLV (TAKARA) according to the manufacturer's instructions. Real-time quantitative PCR was performed on a StepOnePlus real-time PCR system (Applied Biosystems) with Power SYBR Green PCR Master Mix (Applied Biosystems) according to the manufacturer's instructions. Endogenous *GAPDH* mRNA served as an internal standard. To measure the half-lives of two endogenous mRNAs, actinomycin D was added to the culture medium at the concentration of 5 mg ml^–1^, to terminate transcription, and RNA samples were collected at 0, 3 and 9 h post transcription termination. Primers used for detecting endogenous mRNAs by real-time PCR are listed below:

*FBXL19*: 5′-CCATGCTCAGTGGTGTGGTT-3′, 5′-GCAGGCCTTGTAGTCGGTTC-3′

*ZBTB7B*: 5′-GATTCACCAGGAACGACAAGC-3′, 5′-CTGTGTGCAGGTGCATGTGG-3′

*PRR12*: 5′-AATCGGTACCAGCGCCTCTA-3′, 5′-CAGAGCGTCTGACTGGCACT-3′

*GPATCH8*: 5′-TATTGGACACCGCTTACTCCA-3′, 5′-ATGGGATCTGTTCTCCCCTGA-3′

*PCBD2*: 5′-TAAAGCAGCAGGATGGTCGG-3′, 5′-GGACATAAAGCCAAATGCCTGA-3′

*CA5B*: 5′-AACCGAGCCTTGCATCCAC-3′, 5′-CAGGTGGCTGGGTCATAAGAG-3′

*LENG8*: 5′-TGCGAGCAGATGAAGTCGAT-3′, 5′-TCATGGTCACCCTTCTCCAA-3′

*TPRN*: 5′-GATCCGGCTCAGAGGAGAAG-3′, 5′-GCTGGACAGGCCTGAGCTAC-3′

*BCKDK*: 5′-GCCATGAGGATCTCAGACCG-3′, 5′-CGCCACTATGCATGTCCAGA-3′

*ZNF316*: 5′-CCAATTCCCAAGCCCGATCT-3′, 5′-AGAACCAGGCTCCTGTGTAGA-3′

*UQCC3*: 5′-TGCTAAAGGAGATGCCACTG-3′, 5′-CCAACCATCCAGTTCTTCCT-3′

*ABCB9*: 5′-ACTTGCACCGTGTGATCTCC-3′, 5′-ATGATGAAGCCGTGGGCATT-3′

*PSMD5-AS1*: 5′-CAAGGCATCTTCACTGCTGTA-3′, 5′-CCCACTCAGTCCTCTTCCAT-3′

*TMEM184A*: 5′-AGCTTCCTGAGCCTGTGTTT-3′, 5′-ACAAGCAGCTGGACTTGATG-3′

*ZNF646*: 5′-GCTGCAGTTAGGGCCTTGG-3′, 5′-ATGGGGCAACGTAGCAGAAA-3′

*MED13*: 5′-TCATCAAAGAGGGTGATGGA-3′, 5′-ATTCCTGCTCCGCTGTTACT-3′

*CRTC3*: 5′-CTGTCGCGGGTTCAATTTCAG-3′, 5′-GCTTGGTGAAATGACGGCTG-3′

*HPRT1*: 5′-TGACACTGGCAAAACAATGCA-3′, 5′-GGTCCTTTTCACCAGCAAGCT-3′

*18S rRNA*: 5′-CGGCGACGACCCATTCGAAC-3′, 5′-GAATCGAACCCTGATTCCCCGTC-3′.

### Bioinformatics analysis

PA-m^6^A-seq data^55^ (GSE54921) and YTHDF2 PAR-CLIP data^22^ (GSE49339) were downloaded from the Gene Expression Omnibus. After removing adapter sequences, the sequencing reads were mapped to the hg19 genome assembly using Bowtie 2 with the default parameters and the number of reads mapped to each of the m^6^A- and YTHDF2-target genes identified in previous studies^22^,^55^ were counted. m^6^A target genes were sorted by their ratio of reads counted in PA-m^6^A-seq relative to RNA-sequencing data obtained in HeLa cells (that is, in-house sequencing data). YTHDF2 target genes were defined as having at least one peak in two of the three biological replicates of the YTHDF2 PAR-CLIP experiments. In total, 3,352 genes exhibiting both m^6^A modification and YTHDF2 binding, 1,297 genes with m^6^A modification but no YTHDF2 binding and 2,348 genes with YTHDF2 binding but no m^6^A modification were identified.

### Data availability

All data used to obtain the conclusions in this study are presented in the article and the Supplementary Materials. Other data and materials may be requested from the authors.

## Additional information

**How to cite this article:** Du, H. *et al*. YTHDF2 destabilizes m^6^A-containing RNA through direct recruitment of the CCR4–NOT deadenylase complex. *Nat. Commun.* 7:12626 doi: 10.1038/ncomms12626 (2016).

## Supplementary Material

Supplementary Information Supplementary Figures 1-6
