# Supplementary material for: YTHDF2 destabilizes m6A-containing RNA through direct recruitment of the CCR4–NOT deadenylase complex
Source: Nat Commun. 2016 Aug 25;7:12626. doi: 10.1038/ncomms12626 (PMC5007331; doi:10.1038/ncomms12626)
Supplement: Supplementary Information — Supplementary Figures 1-6 [file ncomms12626-s1.pdf]

Supplementary Figures

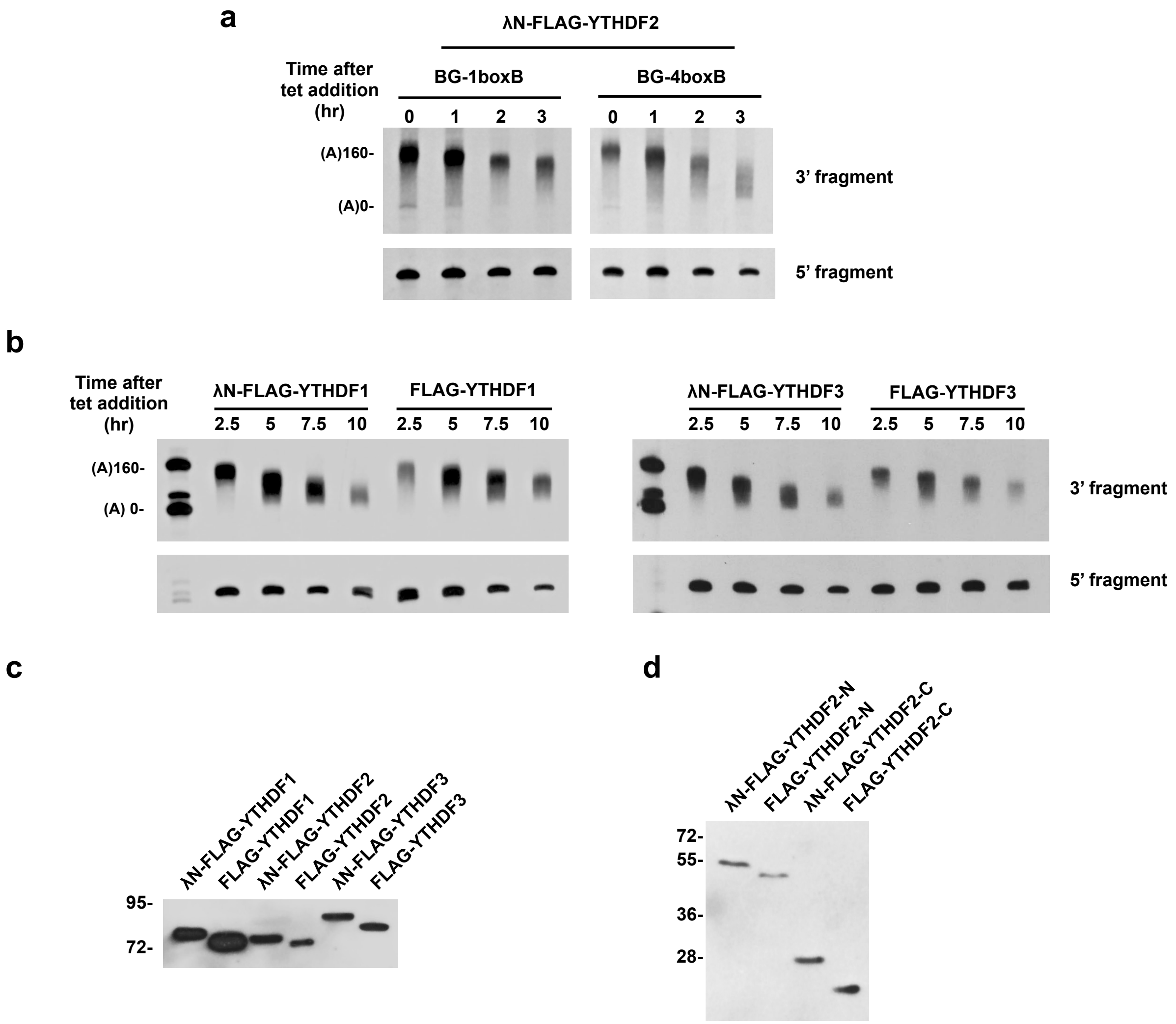

Supplementary Figure 1. YTHDF1 and YTHDF3 also promote deadenylation.
(a) Deadenylation assay of BG-1boxB or BG-4boxB mRNA in the presence of λN-FLAG-YTHDF2. (b) Deadenylation assay of BG-1boxB mRNA in the presence of λN-FLAG-YTHDF1 or FLAG-YTHDF1 and λN-FLAG-YTHDF3 or FLAG-YTHDF3. (c) Immunoblotting assay of ectopically expressed λN-FLAG or FLAG-tagged YTHDF1/2/3. (d) Immunoblotting assay of ectopically expressed λN-FLAG or FLAG-tagged YTHDF2-N/C.

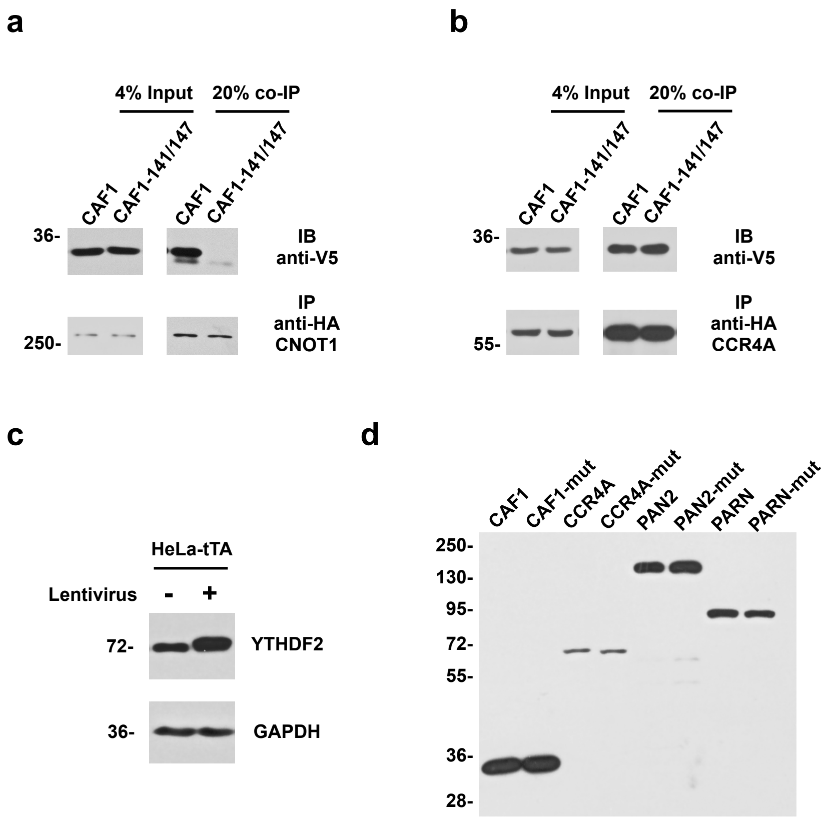

Supplementary Figure 2. The CAF1-141/147 mutant is incapable of interacting with CNOT1. (a) Interaction between CNOT1 and CAF1 or CAF1-141/147, a mutant version of CAF1 with M141K and L147K double substitution. HEK 293 cells were co-transfected with a plasmid encoding HA-tagged CNOT1 and V5-tagged CAF1 or CAF1-141/147. Lysates were subjected to immunoprecipitation by using anti-HA affinity gel. Input and co-purified proteins were blotted by probing with corresponding antibodies. (b) Interaction between CCR4A and CAF1 or CAF1-141/147. HEK 293 cells were co-transfected with a plasmid encoding HA-tagged CCR4A and V5-tagged CAF1 or CAF1-141/147. Immunoprecipitation and immunoblotting were performed as in (a). (c) Immunoblotting assay of YTHDF2 in HeLa-tTA cells with or without stably expressed FLAG-tagged YTHDF2 mediated by lentivirus infection. GAPDH serves as the loading control. (d) Immunoblotting assay of ectopically expressed V5-tagged wild-type or catalytically inactive deadenylases.

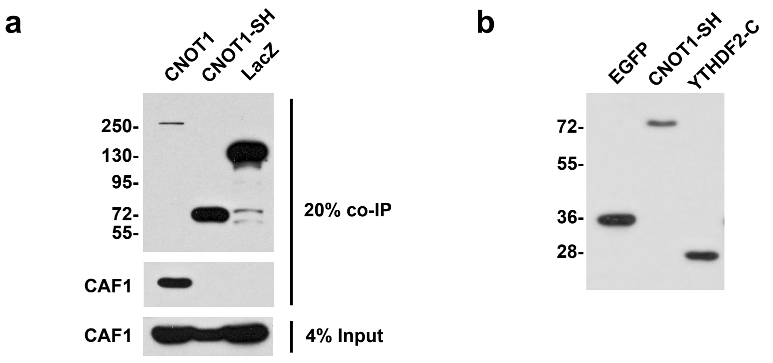

Supplementary Figure 3. The SH domain of CNOT1 is incapable of recruiting CAF1.
(a) Interaction between CAF1 and CNOT1 or CNOT1-SH. HEK 293 cells were co-transfected with a plasmid encoding V5-tagged CAF1 and HA-tagged CNOT1, CNOT1-SH or LacZ (negative control). The HA-tagged proteins were immunoprecipitated using an anti-HA affinity gel. Input and co-purified proteins were detected by corresponding antibodies. (b) Immunoblotting assay of ectopically expressed FLAG-tagged EGFP, YTHDF2-C or CNOT1-SH.

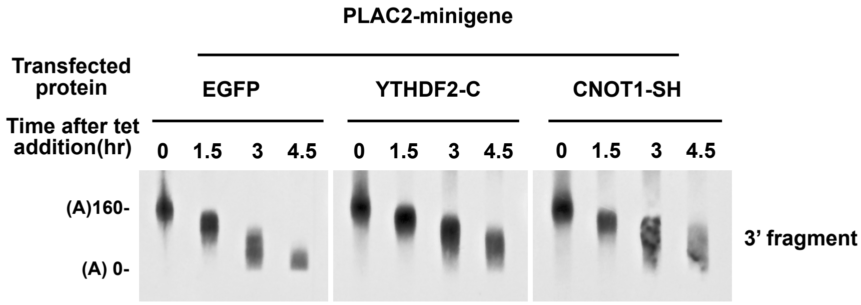

Supplementary Figure 4. *PLAC2* minigene shows decreased deadenylation rate upon YTHDF2-C or CNOT1-SH overexpression.

Deadenylation assay of *PLAC2* minigene reporter upon overexpression of YTHDF2-C, CNOT1-SH or the negative control EGFP**.**

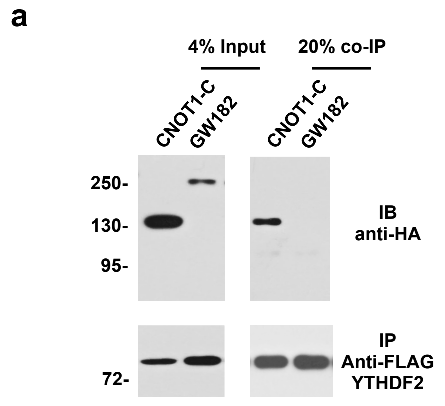

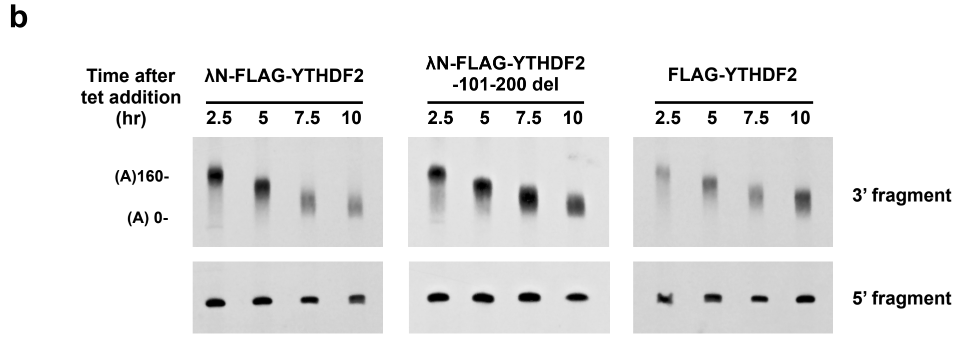

Supplementary Figure 5. GW182 does not exhibit a detectable interaction with YTHDF2 and YTHDF2 mutant lacking aa 101-200 still causes some degree of accelerated deadenylation . (a) Interaction between YTHDF2 and CNOT1-C or GW182. HEK 293 cells were co-transfected with a plasmid encoding FLAG-tagged YTHDF2 and HA-tagged CNOT1-C (aa 1321-2376) or GW182. Lysates were subjected to immunoprecipitation by using anti-FLAG affinity gel. Input and co-purified proteins were blotted by probing with corresponding antibodies. (b) Deadenylation assay of BG-1boxB mRNA in the presence of λN-FLAG-YTHDF2 or λN-FLAG-YTHDF2-101-200 del or FLAG-YTHDF2.

Fig. 1d

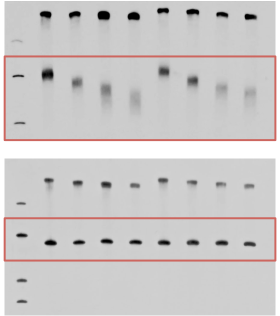

Fig. 2d

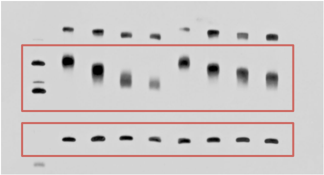

Fig. 3a

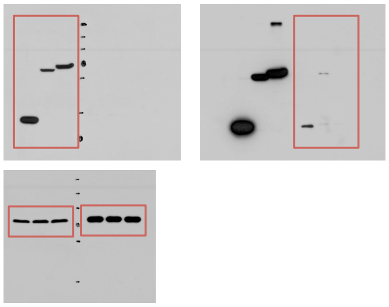

Fig. 3b

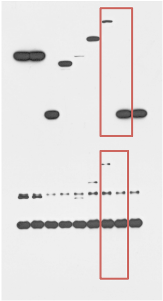

Fig. 3e

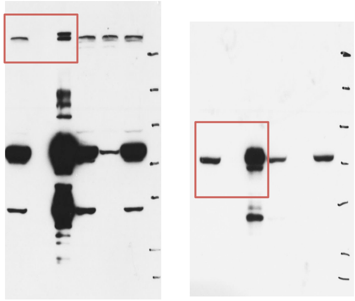

Fig. 4b

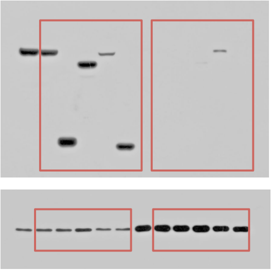

Fig. 4c

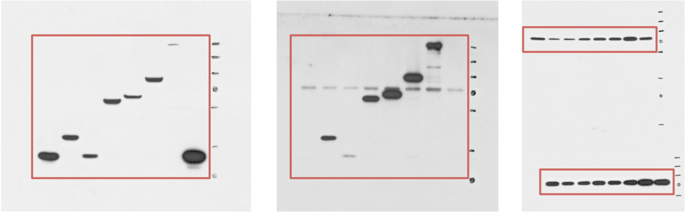

Fig. 5a

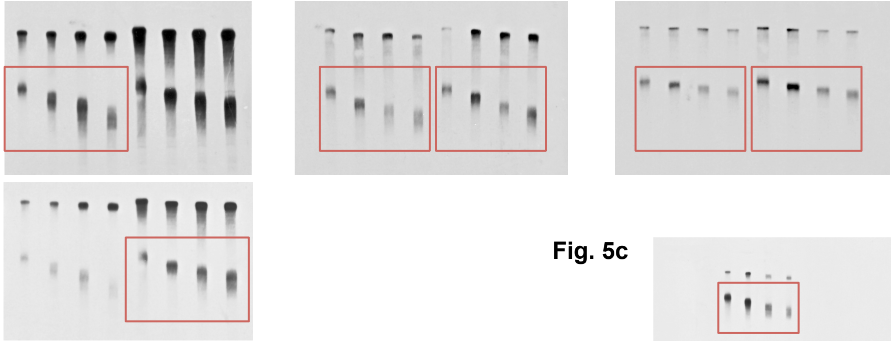

Fig. 5c

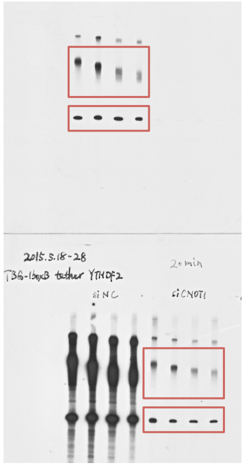

Fig. 5b

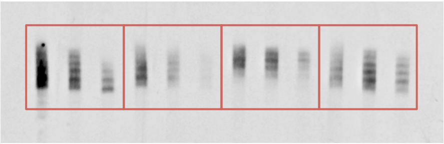

Supplementary Figure 6. Uncropped scans of Northern and Western blots.
